# Kinesiophobia is not required to predict chronic low back pain in workers: a decision curve analysis

**DOI:** 10.1186/s12891-020-3186-8

**Published:** 2020-03-12

**Authors:** A. M. Panken, J. B. Staal, M. W. Heymans

**Affiliations:** 1 Department of Epidemiology and Biostatistics, Amsterdam Public health research Institute, Amsterdam UMC, Vrije Universiteit Amsterdam, Amsterdam, The Netherlands; 2 Physical Therapy Practice Panken, Roermond, The Netherlands; 3 Han University of applied sciences, Nijmegen, The Netherlands

**Keywords:** Low Back pain, Prognosis, Prediction model, Kinesiophobia, Decision curve analysis

## Abstract

**Background:**

Currently used performance measures for discrimination were not informative to determine the clinical benefit of predictor variables. The purpose was to evaluate if a former relevant predictor, kinesiophobia, remained clinically relevant to predict chronic occupational low back pain (LBP) in the light of a novel discriminative performance measure, Decision Curve Analysis (DCA), using the Net Benefit (NB).

**Methods:**

Prospective cohort data (*n* = 170) of two merged randomized trials with workers with LBP on sickleave, treated with Usual Care (UC) were used for the analyses. An existing prediction model for chronic LBP with the variables ‘a clinically relevant change in pain intensity and disability status in the first 3 months’, ‘baseline measured pain intensity’ and ‘kinesiophobia’ was compared with the same model without the variable ‘kinesiophobia’ using the NB and DCA.

**Results:**

Both prediction models showed an equal performance according to the DCA and NB. Between 10 and 95% probability thresholds of chronic LBP risk, both models were of clinically benefit. There were virtually no differences between both models in the improved classification of true positive (TP) patients.

**Conclusions:**

This study showed that the variable kinesiophobia, which was originally included in a prediction model for chronic LBP, was not informative to predict chronic LBP by using DCA. DCA and NB have to be used more often to develop clinically beneficial prediction models in workers because they are more sensitive to evaluate the discriminate ability of prediction models.

## Introduction

The development of prediction models has grown in popularity in Low Back Pain (LBP) research [1–3]. Prediction models can help clinicians as physical therapists in daily practice in making a prognosis and provide an estimate of the probability of persisting symptoms for individual patients [4]. This probability estimate may be an indication for the clinician to adjust the treatment goals to the patient needs.

A recent literature review showed that most prediction models (developed for physical therapists) do not use performance measures that evaluate the clinical usefulness of the models well [5]. Performance measures as Receiver Operating Characteristic Curve (ROC curve), the Area Under this Curve (AUC), sensitivity and specificity, in combination with a low and high risk cutoff point can be used to determine the clinical feasibility of a developed prediction model. However, these measures have shown to be less sensitive in evaluating the added discriminative performance of a predictor and do not provide direct feedback on the number of chronic LBP patients that are correctly classified and are therefore less clinically useful [6–8]. A novel performance measure to evaluate the discriminative ability of a prediction model is Decision Curve Analysis (DCA) and the Net Benefit (NB) [9]. This method is able to identify the number of patients that are better classified and incorporate clinical consequences of using a model, which is useful for clinicians [7]. Furthermore, this novel method is recommended by recent guidelines to develop prediction models (Transparent Reporting of a multivariable prediction model for Individual Prognosis Or Diagnosis (TRIPOD) statement) [10]. Remarkably is that this method has not been used frequently by LBP researchers yet. To our opinion, until now only two studies predicting LBP using DCA, were published [11, 12]. Heymans et al. published a model to predict chronic LBP in workers that included the variables of a ‘clinically relevant decrease in pain intensity and in disability status in the first 3 months’, ‘pain intensity at baseline’ and ‘kinesiophobia’ [13]. The importance of kinesiophobia as a prognostic factor for chronic LBP was suspect. The fear-avoidance model was originally developed to explain the transition from acute to chronic pain [14]. However, there was conflicting evidence in the literature about the clinical usefulness of kinesiophobia as a predictor for chronic LBP. Gheldof et al. stated that, fear of movement measured with the Tampa scale only was a risk factor in case of failure to recovery from short-term LBP [15]. The impact of kinesiophobia on the transition from subacute to chronic LBP was also reported in the studies of Heneweer et al. [16] and Swinkels-Meewisse et al. [17]. Also, Dawson et al. stated that kinesiophobia increased the likelihood of sick leave due to LBP [18]. Furthermore Lakke et al. showed in a synthesis of evidence from systematic reviews (SRs) that kinesiophobia was often included in studies as a possible predictive variable, although this was not always justified [19]. So, the objective of the current study is to evaluate if kinesiophobia is a clinically relevant predictor of chronic LBP in the light of the novel discriminative performance measure, Decision Curve Analysis (DCA), using the Net Benefit (NB) because this measure is more suitable to test the predictive performance of separate predictor variables.

## Methods

### Study design

A prospective cohort study (n = 170) was used by merging data from workers on sick-leave with LBP that received usual care (UC), in two randomised controlled trials (RCTs) [20, 21]. This study was reported in accordance with the strengthening the reporting of observational studies in epidemiology (STROBE) statement [22]. These RCTs were conducted at the same department, within the same time frame, were similar in design, used the same baseline and follow-up variables and the same in/exclusion criteria: patients with non-specific LBP, on sickleave for 4–8 weeks, visited their occupational physician. For detailed information about the data merging process see Heymans et al. [13]. Both RCTs were approved by the medical ethical committee

### Outcome measure

Pain intensity was assessed on a Numerical Rating Scale (NRS) at baseline, 3 and 6 months [23]. The outcome measure chronic LBP (0 = no, 1 = yes) was defined as having a pain intensity score of ≥4 at baseline and ≥ 3 at three and 6 months of follow-up [13, 24].

### Prediction models used in this study

The models were derived from the study published in 2010 [13]. In the current study, the value of the variable ‘kinesiophobia’ was studied comparing the following models.

- *Model 1* consisted of the variables ‘pain intensity at baseline,’ a ‘clinically relevant change in pain intensity [21] and in disability status [25] in the first 3 months’. A clinically relevant change in pain and disability was noted by a change of 3 and 4 points on the NRS and Roland Disability Questionnaire (RDQ) respectively within the first 3 months after the LBP episode [26, 27].
- *Model 2* was model 1, plus an extra variable under study: ‘kinesiophobia’ [28].

### Statistical analysis

A logistic regression model was used to study the relationship between the outcome measure and the aforementioned predictors. In the original paper of Heymans et al. [13], the variables ‘Pain intensity at baseline’ missed 3% of the data, ‘kinesiophobia’ 6,2%, ‘change in pain intensity in the first 3 months’ 19,7% and ‘change in functional status in the first 3 months’ 23,7% missing data. These missing values were replaced by applying multiple imputation (MI) by using the Multiple Imputation by Chained Equations package [29]. For the current study the first imputed dataset (from 10) was used from the original study to evaluate the DCA analysis of the prediction models that compared the inclusion of the kinesiophobia variable. This procedure was followed for practical reasons because if we had used all multiple imputed datasets from the original study, we had to somehow pool all DCA and Net Benefit results when they were applied in each imputed dataset and these pooling methods are not available. Moreover, our study goal was *to compare* DCA and Net Benefit of two prediction models and we think that we were still able to fulfill that goal accurately by using one of the imputed datasets because the regression coefficient estimates in this dataset were strongly comparable to the pooled estimates from the original study. The AUC values (95% Confidence Intervals) of each model were also presented. All statistics were done with R software using Harrell’s rms package.

### Decision curve analysis

Decision curve analysis (DCA) is a method to evaluate the net benefit (NB) of a prediction model across clinicians and patient preferences for accepting the risk of under- or overtreatment [9, 30]. The decision to treat depends on the benefits (effectiveness) and harms (complications, costs) of the treatment. For this, in DCA the ‘probability threshold’ (p_t_) is important: a level of certainty of the outcome above which the patient would choose to be treated. This threshold includes the relative value of the patient for receiving treatment when thinking he/she develops chronic LBP in relation to the value of avoiding treatment thinking he/she will recover from LBP. If the treatment is effective with minimal costs and risk of complications this threshold will be low. On the other hand, if the treatment is associated with high intensity, minimal effect and high costs, the threshold will be high. The net benefit (NB) is calculated by the difference between the expected benefit and harm associated with the treatment. The expected benefit incorporates the number of patients who will correctly develop chronic LBP according to the prediction model and will be treated: the true positive patients (TP). The expected harm incorporates the number of patients who will recover from LBP but would be treated (the false positives = FP) multiplied by a weighting factor based on the patient’s threshold probability. In formula: NB = (TP - *w* FP)/N, where N is the total number of patients and the *w* in the NB formula is described by p_t_/(1 - *p*_t_). For example, a physiotherapist uses a prediction model to determine the probability of developing chronic LBP and wonders if a patient with a probability of 30% according to the model has to be treated by an exercise program. In formula: w = 0.3/(1–0.3) = 0.43, which means that the number of FP patients in the NB formula gets less weight and that unnecessary treatment is less important than missing treatment (because w is multiplied by FP). When the patient worries about the LBP and/or the treatment is effective, cheap and not intensive, a physical therapist could decide to treat the patient at this low risk of chronic LBP [21]. A physical therapist that uses a higher p_t_ of 70% (w = 0.7/0.3 = 2.33), assumes FP decisions more harmful. This may play a role in case of intensive and costly exercise programs [21]. Because patients and clinicians may value harms and benefits differently, the NB can be calculated for different value of p_t_’s and compared to the NB of treating all patients (assuming everybody develops chronic LBP and needs treatment) or treating no patients (assuming nobody develops chronic LBP). This can be graphically depicted by making a decision curve. A higher NB value means that the model will be more clinically useful as indicated by the higher number of TP patients that are identified. Further, the NB of prediction model 1 can be compared to the NB of prediction model 2 at each level of p_t_ and it can be evaluated if the variable ‘kinesiophobia’ is needed to improve the predictive performance of the prediction model. This will be further clarified and explored in the results section.

## Results

Table 1 showed the patients characteristics of all occupational LBP patients (*n* = 170) of which 91 patients developed chronic LBP (53.5%).

**Table 1:** Patient characteristics at baseline (*n* = 170) of the LBP patients

	Value	Observed range
Age (mean years ± SD)	39.4 (9.2)	18–59
Gender (number male (%))	146 (85.8)	–
Pain intensity (NRS) (mean ± SD)	6.45 (1.9)	0–10
Kinesiophobia (mean ± SD)	39.6 (6.8)	23–62
Change in pain intensity (%)	100 (58.8)	–
Change in functional disability (%)	110 (64.7)	–
Chronic Low Back Pain (%)	91 (53.5)	–

*SD* standard deviation, *NRS* numerical rating scale

Table 2 showed the strength of the relationships of the variables in the two prediction models.

**Table 2:** Odds ratios (OR) of the 2 prediction models compared

Prediction Models	OR (95% CI)	AUC (95% CI)
Model 1		0.858 (0.780;0.917)
Change in pain intensity	4.09 (0.83;20.11)	
Change in functional disability	8.57 (1.59;46.25)	
Pain Intensity at baseline	2.10 (1.59;2.77)	
Model 2		0.862 (0.805;0.920)
Change in pain intensity	3.64 (0.76;17.47)	
Change in functional disability	10.43 (1.92;56.72)	
Pain Intensity at baseline	2.01 (1.52;2.66)	
Kinesiophobia	1.05 (0.99;1.11)	

Model 2 (with the added variable ‘kinesiophobia’) did not perform better compared to Model 1 given the AUC of 0.862 compared to the AUC of model 1 of 0.858. Further, the strength of all variables in the models remained the same and the variable ‘kinesiophobia’ in Model 2 showed an insignificant OR of 1.05 with 95% CI of 0.99–1.11.

### Decision curve analysis

#### Comparing both prediction models at one threshold probability

First, our comparison started with an example of the calculation of the NB for prediction model 1, the model without the variable ‘kinesiophobia’ at a p_t_ of 30% by using Table 3. At a p_t_ of 30% the number of TP patients according to the prediction model was 86 and the number of FPs was 38. With a total number of patients of 170 the NB = 86/170 – (38/170 x (0.3/0.7)) = 0.410. This NB meant that a net 41 TP patients per 100 patients was identified, compared to assuming that all patients did not develop chronic LBP, at the same number of FP patients. The calculation of the NB for prediction model 2, that also included the variable ‘kinesiophobia’, was 84/170 – (33/170 x (0.3/0.7)) = 0.411. The interpretation of this NB was that a net 41 TP patients per 100 patients was identified, compared to assuming all patients were negative, at the same number of FP patients. At this level of p_t_ the NB of both models were similar and the prediction was not improved by including the variable ‘kinesiophobia’.

**Table 3:** Relationship between chronic LBP and results of a prediction models with a predicted probability of chronic LBP of 30%

Model 1	Chronic LBP	No Chronic LBP	Total	NB	N
Probability of chronic LBP ≥ 30%	86	38	124	0.410	41
Probability of chronic LBP < 30%	5	41	46		
	91	79	170		
Model 2	Chronic LBP	No Chronic LBP	Total	NB	n
Probability of chronic LBP ≥ 30%	84	33	117	0.411	41
Probability of chronic LBP < 30%	7	46	53		
	91	79	170		

*NB* Net Benefit at probability threshold (p_t_) of 30%

*N* amount of true positive identified patients per 100 patients, compared to assuming that all patients did not develop chronic LBP, at the same number of false positive patients

When it was assumed that all patients were positive and developed chronic LBP, the NB was calculated as NB = 91/170 – (79/170 * (0.3 / 0.7)) = 0.336. This value was lower as the NB of prediction model 1 and 2 above, which meant that both the prediction models were more of clinical benefit at a p_t_ of 30% than just assuming everybody had LBP and treat them accordingly. The difference in NB between both prediction models and assuming that all patients were positive was (NB_model_ – NB_treat all_) * 100 = (0.411–0.336) * 100 = 7.5. This meant that a net 8 TP patients was identified by using the prediction models compared to treating all patients, without an increase in de number of FP patients. Comparing prediction models 1 and 2, where model 2 contained the extra predictor ‘kinesiophobia’, at a p_t_ of 30%, there was no difference in NB between these models.

#### Comparing both prediction models at various threshold probabilities - the decision curve

**Fig. 1:**
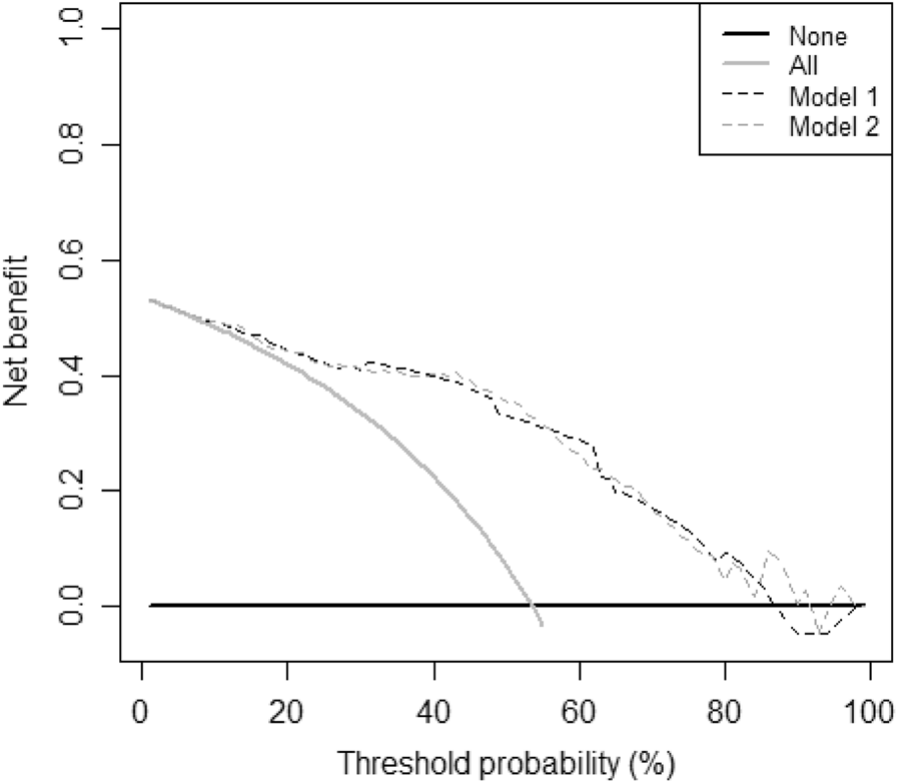
Decision curves of the prediction Models 1 and 2 to predict chronic LBP. Y-axis is Net Benefit and x-axis is threshold probability p_t_. Dotted black line belongs to the Net Benefit of prediction Model 1, dotted grey line to Model 2. The black line is the Net Benefit when all patients are assumed negative and the grey line is the Net Benefit when everybody is assumed positive and would be treated. * The Net Benefit of the model around 90% is sometimes negative due to random noise [9]

On the decision curve the NB of the prediction models (y-axis) according to the various threshold probabilities p_t_ (x-axis) was plotted. The NBs of the prediction models 1 and 2 were shown in Fig. 1.

It could be seen in Fig. 1 that there were virtually no differences between prediction models 1 and 2 in NBs, i.e. adding ‘kinesiophobia’ did not increase the NB over the whole range of p_t_’s. Table 4 showed the differences in NBs and the improvement in the detection of TP patients of prediction model 2 compared to model 1 at different p_t_’s. Both models had a higher NB compared to treating everybody over the range of p_t_’s of 10% to just over 50%. This improvement resulted in the identification of more TP patients for both models compared to treating everybody. Further, prediction model 1 and 2 had slightly the same NBs. Over the whole range of p_t_’s they alternated in the identification of marginally more TP patients. This meant that the variable ‘kinesiophobia’ was not necessary to improve the prediction of chronic LBP.

**Table 4:** Net Benefits of prediction Models 1 and 2 compared to the NB of treating nobody or everybody and the consequences for the number of TPs at probability thresholds ranging from 5 to 60%

Pt^a^	NB Model 1^b^	NB Model 2 ^b^	NB treat all	TP M1 vs all^c^	TP M2 vs all^c^	TP M2 vs M1^d^
0.05	0.513	0.513	0.511	0	0	0
0.1	0.493	0.493	0.484	1	1	0
0.2	0.444	0.441	0.419	2	2	0
0.3	0.410	0.411	0.336	7	7	0
0.4	0.398	0.402	0.225	17	18	0
0.5	0.329	0.353	0.071	26	28	2
0.6^e^	0.288	0.265	−0.162	45	43	−2

^a^P_t_ is threshold probability

^b^ NB is Net Benefit of the Model compared to assuming everybody does not develop chronic LBP

^c^ TP is the increase in number of true positive patients when the Model is compared to assuming everyone has chronic LBP

^d^ TP is the increase in number of true positive patients when Model 2 is compared to Model 1

^e^ Only comparison until 60% was feasible because the NB for treat all was negative at higher percentages

## Discussion

### Main findings

In 2010 it was shown by Heymans et al. [13] that a prediction model including ‘pain intensity at baseline’,

‘kinesiophobia’ and a ‘clinically relevant decrease in pain intensity and in disability status in the first 3 months’ predicted chronic LBP well. The finding that changes in the initial LBP and functional status period were relevant to predict chronic LBP later in time was demonstrated in more studies [31–34]. In the current study it was shown that the variable ‘kinesiophobia’ was not required to predict chronic occupational LBP in workers by using the novel performance measures DCA and NB.

### Decision curve analysis

Often it was not totally clear when a prediction model was of benefit for clinicians and/or patients. It made therefore sense to evaluate the clinical value of the model at different levels of threshold probabilities by using the NB. For example, when patients worried about their LBP, the physical therapist might want to know if the model was still of benefit at low risk probabilities. The patient might then be successfully referred to a low intensive and cheap intervention program [21]. To know at which probability threshold the prediction model was clinically useful, we had to know what kind of risk probabilities physical therapists used in practice and what kind of harm and benefits were acceptable for physical therapists and patients. That was challenging for LBP because physical therapists might think differently about LBP and the consequences of treatment for their patients.

### Strengths and limitations

Performance measures as sensitivity and specificity could be used to determine the discriminative ability of a prediction model. However, these measures are less suitable to test the predictive performance of separate predictor variables [6]. Sensitivity or specificity may decrease even when the ROC curve of one model uniformly dominates the ROC curve of the other model. The NRI and decision-analytic measures will agree in sign in reasonable scenarios [8].

Therefore, the sensitivity, specificity and ROC curve were not considered in this study. A limitation could be that our model was only internally validated, however internal validation was sufficient to allow the use of DCA [35]. Our definition of chronic LBP was not applicable to all types of chronic LBP patients in practice. For example, the prognosis of patients with pain free episodes (not identified by our definition) might be determined by other variables for chronic LBP. Furthermore, our definition was determined on the level of pain intensity. It had been argued that chronic LBP may not only be based on pain intensity but also on limitations in function [36]. However, recent studies showed that LBP pathways were linked to functional disability in such a way that if one knew the level of pain intensity, also the level of functional limitations could be determined and vice versa [34]. Another limitation is that data were used from RCTs rather than observational studies. RCTs apply strict inclusion and exclusion criteria that can for example result in a more homogeneous patient population that may affect the performances of the prediction model [4]. Consequently, this may hamper the generalization of results to a group of patients that is seen in daily practice.

## Conclusion

In our study ‘kinesiophobia’ (measured by the TS) seemed not required to improve the prediction of chronic occupational LBP and that it was not needed to adapt the treatment strategy. The performance measures NRI and DCA were not used in LBP research and practice yet. Why these measures were not used is unclear. Perhaps because the most published articles concerning the NRI and NB methods were published in methodological or statistical oriented journals. Although, the use of probability thresholds was mentioned before for physical therapists within the context of clinical decision making [37, 38]. The DCA gave the best insight in the clinical usefulness of prediction models for physical therapists. They could translate clinical usefulness and benefits in terms of number of TP patients that were identified, which is attractive for healthcare professionals and their patients especially in the light of making good treatment decisions.

## Data Availability

The datasets used and/or analysed during the current study are available from the first author on reasonable request.

## Abbreviations

AUC: Area under this curve
DCA: Decision curve analysis
FP: False positive
LBP: Low back pain
NB: Net benefit
NRS: Numerical rating scale
p_t_: Probability threshold
RCT: Randomized clinical trial
ROC: Receiver operating characteristic
STROBE: Strengthening the reporting of observational studies in epidemiology
TP: True positive
TRIPOD: Transparent Reporting of a multivariable prediction model for individual prognosis or diagnosis
UC: Usual care
